# MID-LIFE AND LATE-LIFE ENVIRONMENTAL EXPOSURES ON DEMENTIA RISK: THE MODIFYING EFFECTS OF APOE

**DOI:** 10.1093/geroni/igae098.1358

**Published:** 2024-12-31

**Authors:** Victoria Williams, Ralph Trane, Kamil Sicinski, Pamela Herd, Michal Engleman, Sanjay Asthana

**Affiliations:** University of Wisconsin Madison School of Medicine and Public Health, Madison, Wisconsin, United States; University of Wisconsin Madison, Madison, Wisconsin, United States; University of Wisconsin Madison, Madison, Wisconsin, United States; Georgetown University, Washington, District of Columbia, United States; University of Wisconsin Madison, Madison, Wisconsin, United States; University of Wisconsin Madison, Madison, Wisconsin, United States

## Abstract

Increasing evidence supports a deleterious effect of late-life air pollution exposure on dementia risk that is exacerbated among older ApolipoproteinE-4 (ApoE-4) carriers. However, the protracted effects of mid-life occupational exposures on dementia outcomes, and potential interactions with ApoE, are unknown. Using prospective life course data from 3,814 participants in the Wisconsin Longitudinal Study (WLS), we evaluated the effects of mid-life occupational exposure to respiratory hazards and late-life air pollution exposures on dementia risk, and whether these associations varied by ApoE-4 status. Mid-life occupational exposure was associated with 1.64 times greater odds of all-cause dementia, but only among ApoE-4 noncarriers. Conversely, late-life air pollution exposure (higher levels of particulate matter (PM2.5) and lower levels of ozone) associated with increased dementia risk only within ApoE-4 carriers. Findings suggest that ApoE-4 differentially impacts the association between environmental exposures and dementia risk based on timing of exposure across the life course – supporting ApoE as a pleiotropic modifying gene. While ApoE-4 appears protective against the adverse effects of hazardous respiratory exposures in mid-life, it increases risk for dementia associated with environmental exposures in late-life.

